# The Anatomist's Vade-Mecum: A System of Human Anatomy

**Published:** 1845-01

**Authors:** 


Art. V.—*The Anatomist's Vade-Mecum: a System of Human Anatomy.* By Erasmus Wilson. With 186 Illustrations, by Bagg. Third Edition.—*London,* 1845. 8vo, pp. 648.

We said so much in praise of the beauty and goodness of this book on two former occasions, that we feel at a loss what to add on its third appearance on our stage. And yet it merits additional commendation, as it now presents us with all its old excellencies, and many new. Among the last, we observe the following: A description of the development of bones, p. 4; the structure of ivory, p. 65; the general anatomy of cartilage and fibrous tissue, pp. 112-17; the structure of muscular fibre, p. 163; the structure of nerve, p. 401. These subjects are all beautifully illustrated by figures "drawn from the microscope by the author himself, with the aid of the camera lucida." The new figures, twenty in number, as well as all the rest are admirable. One great merit of the 'Vade-Mecum' is, that it comprises good general as well as descriptive anatomy. The following interpretation of well-known appearances in the conformation of the vertebral column, is as beautiful as ingenious.

"The transverse processes consist essentially of two parts, the anterior of which in the dorsal region is the rib, while the posterior retains the name of the transverse process. In the cervical region these two elements are quite apparent, both by their different points of attachment to the vertebra, and by the vertebral foramen which divides them at their base. In the lumbar region the so-called transverse processes are, in reality, lumbar ribs, while the transverse processes will be found behind them in a rudimentary state, developed like the true transverse processes in the cervical region, from the superior articular processes. When the anterior and posterior transverse processes are examined in relation with each other, they will be observed to converge; and if the latter were prolonged they would unite as in the cervical region and enclose a foramen, or they would rest in contact as in the dorsal region, or become consolidated as in the formation of the sacrum. Moreover, the posterior transverse processes are directed upwards, and if they were prolonged, they would come into contact with a small tubercle which is found at the base of the posterior transverse process, (in strongly marked vertebræ) in the vertebra above. This junction would form a posterior intervertebral foramen, as actually occurs in the sacrum. In brief, the lumbar vertebræ exhibit those transitional changes which are calculated, by an easy gradation, to convert separate vertebræ into a solid bone. The transverse processes of the eleventh and twelfth dorsal vertebræ are very interesting in a transcendental point of view, as exhibiting a tendency which exists obscurely in all the rest, namely, to trifurcate. Now supposing these three branches to be lengthened in order to fulfil their purposes, the anterior would constitute the articulation or union with a rib, while the superior and inferior would join similar branches in the vertebra above and below, and so form the posterior intervertebral foramen." (p. 16.)

